# Supplementary material for: Shape-memory polyurethanes for polar wearables with ultrasensitive multi-monitoring
Source: Nat Commun. 2025 Dec 11;16:11329. doi: 10.1038/s41467-025-66422-3 (PMC12722725; doi:10.1038/s41467-025-66422-3)
Supplement: Supplementary file 1 — Supplementary Information [file 41467_2025_66422_MOESM1_ESM.pdf]

Supplementary Information

Shape-Memory Polyurethanes for Polar Wearables with Ultrasensitive Multi-Monitoring

Tianze Chen, Jing Xu, Chongyang Wang, Xinrui Zhang*, Tingmei Wang, Qihua Wang*

T. Chen, J. Xu, C. Wang, X. Zhang, T. Wang, Q. Wang
State Key Laboratory of Solid Lubrication
Lanzhou Institute of Chemical Physics
Chinese Academy of Sciences
Lanzhou 730000, China
E-mail: xruiz@licp.cas.cn; jingxu@licp.cas.cn

T. Chen, C. Wang, J. Xu
College of Materials Science and Opto-Electronic Technology
University of Chinese Academy of Sciences
Beijing 100049, China

Table of Contents

Supplementary Methods ………… S3

Supplementary Discussion ………… S10

Supplementary Figures ………… S13

Supplementary Tables ………… S24

Supplementary References ………… S26

Supplementary Methods

Materials

Polycarbonate diol (PCDL, M_n = 2000 Da, 99%) was purchased from Liduo Chemical Co., Ltd. (Jining, China). Isophorone diisocyanate (IPDI, 99%), malonic acid dihydrazide (MDH, 97%) and dibutyltin dilaurate (DBTDL, 98%) were purchased from Energy Chemical Co., Ltd. (China). 2,2′-Bis(trifluoromethyl) benzidine (TFMB,98%), (2-hydroxyethyl) trimethylammonium bromide (TLB, 98%) and 1-ethyl-3-methylimidazolium bis(trifluoromethanesulfonyl) imide ($[EMIM]^+$ $[TFSI]^-$, 99%) were purchased from TCI (Shanghai) Development Co., Ltd. *N*, *N*-Dimethylformamide (DMF, 99.5%) was purchased from Rionlon Bohua Pharmaceutical & Chemical Co., Ltd. (Tianjin, China). All other chemicals and solvents were of analytical grade and used without further purification.

Synthesis of the PUFTx Elastomer

For the synthesis of the $PUFT_1$: PCDL (M_n = 2000 Da, 5.0 mmol, 10.00 g) was mechanically stirred at 110 °C for 2 h under nitrogen (The following operations are carried out under nitrogen atmosphere if not otherwise specified.) in a three-necked round-bottom flask to remove residual moisture, cooled to 80 °C, and supplemented with a solution of IPDI (10.0 mmol, 2.25 g) and DBTDL (0.02 g) in DMF (40 mL). The mixture was stirred at 80 °C for 3 h, and the resulting prepolymer solution was supplemented with MDH (4.0 mmol, 0.53 g) and TFMB (0.5 mmol, 0.16 g). The mixture was heated at 80 °C for 2 h to a chain extension reaction, then supplemented with TLB (1

mmol, 0.18 g), further heated at 80 °C for 5 h, until the reaction was completed. The resulting liquid was poured into clean Teflon molds, and the samples were vacuum-dried at 80 °C for 48 h. The product was named $PUFT_1$. Following the same procedure for $PUFT_x$ were synthesized by changing the feed mass of MDH, TFMB and TLB. The mixing ratios used to prepare $PUFT_x$ are given in the Supplementary Table S4.

Synthesis of the $PUFT_x$-*y*IL Elastomer

For the synthesis of the $PUFT_2$-50%IL: $PUFT_2$ (2.00 g) was dissolved in DMF (40 mL) and $[EMIM]^+$ $[TFSI]^-$ (2.00g) was added after the dissolution was completed. After 1 hour of stirring to mix well, the mixture solution was poured into clean Teflon molds, and the samples were vacuum-dried at 80 °C for 48 h. The product was named $PUFT_2$-50%IL.

Characterizations

Thermogravimetric analysis (TGA) was evaluated in an SDT 650 synchronous thermal analyzer (TA, America) in the range of 25 to 800 ℃ under a nitrogen flow (100 mL min^{-1}) at a heating rate of 10 ℃ min^{-1}.

Differential scanning calorimetry (DSC) measurements (DSC STA449F3, Netzsch, Germany) were performed in a flow of nitrogen. The samples (5-6 mg) were heated to 150 °C, cooled to -100 °C, and reheated to 150 °C at a rate of 10 °C min^{-1}.

X-ray diffraction (XRD) measurements were characterized on a Bruker D8 Advance (BRUKER, Germany) using Cu-Kα radiation with λ = 1.5418 Å, and the data were recorded in a range from 5° to 90° at a scanning speed of 5° min^{-1}.

Fourier transform infrared (FT-IR) spectra was recorded on a Nicolet iS20 (Thermo Scientific, America) spectrometer from 500 to 4000 cm^{-1} in attenuated total reflectance (ATR) mode.

Hydrogen proton nuclear magnetic resonance (^{1}H NMR) spectra were measured on an AVANCE NEO 400MHz spectrometer (Bruker, Germany), using $CDCl_3$ as solvent.

Raman spectroscopy was recorded using a Raman spectrometer (LabRAM HR Evolution, HORIBA), with the laser power of 25 mW for 532 nm laser excitation.

X-ray Photoelectron Spectroscopy (XPS) was carried out on K-Alpha (Thermo Scientific, America), excited by an Al Kα (hv = 1486.6 eV) Mono X-ray with a beam spot of 400 μm.

Time-of-flight secondary ion mass spectrometry (ToF-SIMS) measurements were conducted utilizing a PHI nanoTOF II Time-of-Flight SIMS (ULVAC-PHI. INC, JAPAN) instrument. Ion mass spectrometry images were acquired by sputtering the sample surface using a 30 keV Bi_3^{++} ion beam in high mass resolution mode over a surface area of 200 μm × 200 μm of the test sample.

The microphase structure of the samples was detected by an atomic force microscope (AFM, Bruker Dimension Icon, Germany) in tapping mode with a scan area of 0.6 μm × 0.6 μm.

Thermomechanical properties were characterized using a dynamic mechanical analyzer (TA Q850 DMA). To that end, all samples were cut into rectangles (20 × 4 × (0.2–0.5) mm) and heated from -120 to 100 °C with a heating rate of 5 °C min^{-1} and a

frequency of 1 Hz.

Tensile tests

Stress-strain curves were recorded for dumbbell-shaped specimens (30 mm × 2 mm × 0.5 mm) at different temperatures and a tensile rate of 10 mm min^{-1} using an electronic universal tensile testing machine AG-X (Shimadzu, Japan). Each sample was tested three times to produce a reliable average.

Toughness (τ) was calculated by integrating the area under the stress–strain curve using Equation 1:

$$\tau = \int_{\varepsilon=0}^{\varepsilon=\varepsilon_{max}} \sigma \, d\varepsilon \,, \quad (1)$$

where σ is the stress, ε is the strain, and $\varepsilon_{\max}$ is the elongation at break.

Cyclic tensile tests

During cyclic tensile tests, the sample was sequentially stretched from 0% to 800% strain without any rest at a constant rate of 10 mm min^{-1} at room temperature.

The dissipated energy and energy dissipation efficiency were calculated using Equations 2 and 3, respectively:

$$\text{Dissipated energy} = S_s - S_r, \quad (2)$$

$$\text{Energy dissipation efficiency} = \frac{S_s - S_r}{S_s} \times 100\%, \quad (3)$$

where S_s is the area under the stretching (loading) curve on the stress–strain graph, and S_r is the area under the retraction (unloading) curve.

Small-angle X-ray scattering (SAXS) measurements

SAXS data was collected using a Xeuss 3.0 instrument (Xenocs, France) equipped

with a semiconductor detector, 30 W phototube, and Cu Kα radiation source (λ = 1.5418 Å). SAXS patterns were acquired in the sample center within 600 s at a sample-to-detector distance of 1000.0 mm and background-corrected. The periodicity of the phase-separated domains (R_d) was calculated using Equation 4:

$$R_d = \frac{2\pi}{q_{max}} \tag{4}$$

where q_{max} represents the scattering vector at which *I* reaches its maximum value.

Electrochemical characterization

Various electrochemical measurements were performed on a Zennium XC electrochemical workstation (Zahner, Germany).

Linear sweep voltammetry (LSV) curves were obtained by measuring at room temperature at a rate of 10 mv s^{-1} over a test range of 0-6V.

Electrochemical impedance spectroscopy (EIS) was performed in the frequency range of 10^{-2} Hz to 10^{5} Hz with an AC amplitude of 10 mV at room temperature. The Ionic conductivity (σ, S cm^{-1}) was calculated by Equation 5:

$$\sigma = \frac{L}{A \times R_b} \tag{5}$$

where L and A correspond to the thickness and area of the samples between the two stainless steel electrodes, and R_b is the bulk resistance.

Shape memory tests

The shape memory properties of $PUFT_2$-50%IL were characterized using a Q850 DMA (TA, USA) at strain mode. The test procedure was as follows: The initial strain (denoted as ε_1) was applied to the specimen at 25°C and kept for 5 min. Then cooled to

-80°C while remaining the strain, and held at this temperature for 10 min to fix the temporary shape. Then kept at -80°C and unloading the force back to 0.001 N (a small preload) for 5 min to obtain the temporary shape, at which time the length of the sample was ε_2, and finally heated again to 25°C and held for 20 min to return to the shape, at which time the sample length was ε_3.

The shape memory recovery rate (R_r) and fixation rate (R_f) were defined by Equations 6 and 7:

$$R_r(N) = \frac{\varepsilon_1 - \varepsilon_3(N)}{\varepsilon_1 - \varepsilon_3(N-1)} \tag{6}$$

$$R_f(N) = \frac{\varepsilon_2(N)}{\varepsilon_1} \tag{7}$$

where N represents the Nth cycle in the shape memory cycle process.

Low-temperature electrical experiments

EIS at low- temperatures was performed in the frequency range of 10^{-2} Hz to 10^{5} Hz with an AC amplitude of 10 mV using a CHIE660E electrochemical workstation (Shanghai ChenHua, China).

Resistance measurements were performed using a Physical Property Measurement System (PPMS, Quantum Design, America). The relative resistance change ($\Delta R/R_0$) was calculated by the Equation 8:

$$\frac{\Delta R}{R_0} = \frac{R - R_0}{R_0} \tag{8}$$

where R and R_0 represent the resistance under different temperatures and the initial resistance.

Sensor performance

The electrodes were connected to the ends of rectangular elastomers, which were stretched at room temperature by a precision universal testing machine.

The specimen was fitted to the part of the human body to be tested and the ends were connected to an Arduino board to obtain a resistance signal through a set program.

A development microcontroller kit was used to measure ECG signals in a three-electrode configuration in conjunction with the Arduino software. To detect the ECG signals, the two working electrodes were attached to the inner wrist while the reference electrode was attached to the ulnar styloid.

Informed signed consent was obtained from volunteers for all tests with human contact.

Self-healing properties

To effectively assess the self-healing properties, all samples were cut into two pieces in the middle and then healed in the same environments at various times. Considering that self-healing ability was often not determined by an individual factor, the η values were calculated by Equation 9:

$$\eta = \frac{P_{healed}}{P_{original}} \times 100\% \tag{9}$$

where P stands for tensile strength, elongation, or toughness.

Supplementary discussion

Quantum chemical calculation

All calculations were carried out with the Gaussian 16, C01 software package[1]. The Becke's three-parameter hybrid exchange functionals and the Lee-Yang-Parr correlation functional (B3LYP) was adopted for all calculations in combination with the D3 version of Grimme's dispersion with Becke-Johnson damping (DFT-D3BJ)[2,3]. For geometry optimization and frequency calculations, the 6-31+G (d, p) basis set was used[4,5]. The singlet point energy calculations were performed with 6-311+G (d, p) basis set[4,6]. The interaction energy of $[EMIM]^{+}[TFSI]^{-}$ were calculated by the following Equation 10:

$$E_{Bind(IL)} = E_{IL} - (E_{+} + E_{-}) \tag{10}$$

where $E_{Bind(IL)}$ denotes the interaction energy of $[EMIM]^{+}[TFSI]^{-}$ in the absence of polymer chains, E_{IL}, E_{+} and E_{-} correspond to the energies of $[EMIM]^{+}[TFSI]^{-}$, isolated $[EMIM]^{+}$ and isolated $[TFSI]^{-}$.

The interaction energy of $[EMIM]^{+}[TFSI]^{-}$ under polymer chain influence were calculated by the following Equation 11:

$$E_{Bind(Complex)} = E_{Complex} - (E_{+} + E_{-} + E_{TFMB} + E_{TLB}) \tag{11}$$

where $E_{Bind(Comples)}$ denotes the interaction energy of $[EMIM]^{+}[TFSI]^{-}$ in the presence of polymer chains, $E_{Complex}$, E_{+}, E_{-}, E_{TFMB} and E_{TLB} correspond to the energies of this system, isolated $[EMIM]^{+}$, isolated $[TFSI]^{-}$, isolated TFMB part and isolated TLB part.

Modeling and simulation detail of MD simulation

In this work, we employed molecular dynamics methods to simulate the ionic conductivity of polymer systems. All molecular dynamics simulations were conducted using the LAMMPS software package with a time step of 1 femtosecond[7]. Initially, we modeled the molecular structures of Polymer 1, Polymer 2, $[EMIM]^+$ ions, and $[TFSI]^-$ ions separately. For System 1, we incorporated 24 Polymer 1 chains, 286 $[EMIM]^+$ ions, and 334 $[TFSI]^-$ ions into the simulation system. For System 2, we included 36 Polymer 2 chains, 277 $[EMIM]^+$ ions, and 349 $[TFSI]^-$ ions. The selection of ion numbers was based on ensuring charge balance and maintaining a mass ratio of 50% between $[EMIM]^+$ and $[TFSI]^-$ ions. We utilized the GAFF potential to describe the polymer molecules and ions, and employed Lennard-Jones and Coulomb potentials to describe intermolecular interactions[8].

Initially, the simulation systems underwent structural local relaxation. Subsequently, using the NPT ensemble, we controlled the temperature at 300K and pressure at 1 atmosphere with a Nose–Hoover thermostat, running relaxation simulations for 1 nanosecond until the systems reached a steady state. The time evolution of the average radius of gyration for the polymer molecules, $[EMIM]^+$, and $[TFSI]^-$ was analyzed during the relaxation process to confirm that the system had reached equilibrium. Following this, over a period of 5 nanoseconds, the systems were uniformly stretched in the x direction to twice their original length, while maintaining a pressure of 1 atmosphere in the y and z directions. To maintain stable box dimensions, we constrained the dimensions of y and z

directions to remain consistent. Then, over another 5 nanoseconds, the systems were compressed back to their original length in the x-direction, again maintaining a pressure of 1 atmosphere in the y and z directions. For the structures before stretching, after stretching, and after compression, we conducted 15-nanosecond simulations under the NVT ensemble, recording atomic configurations every 10 picoseconds. From these atomic configurations, we calculated the mean square displacement (MSD) of the $[EMIM]^+$ and $[TFSI]^-$ ions.

The MSD is defined as the average of the squared distances that the particle has traveled over a time interval (*t*). The MSD is given by the Equation 12:

$$MSD(t) = \frac{1}{N}\sum_{i=1}^{N} \langle |r_i(t) - r_i(0)| \rangle \tag{12}$$

where *N* is the number of particles, $r_i(t)$ is the position of the *i*-th particle at time *t*, $\langle\ \rangle$ denotes the ensemble average.

The diffusion coefficient (*D*) is obtained from the long-time limit of the MSD, calculated by the Equation 13:

$$D = \lim_{t\to\infty} \frac{MSD(t)}{6t} \tag{13}$$

The ionic conductivity (σ) is given by the Equation 14:

$$\sigma = \frac{q^2 c}{k_B T} D \tag{14}$$

where q is the charge of the ion, *c* is the concentration of ions, k_B is the Boltzmann constant, *T* is the temperature in Kelvin.

Supplementary Figures

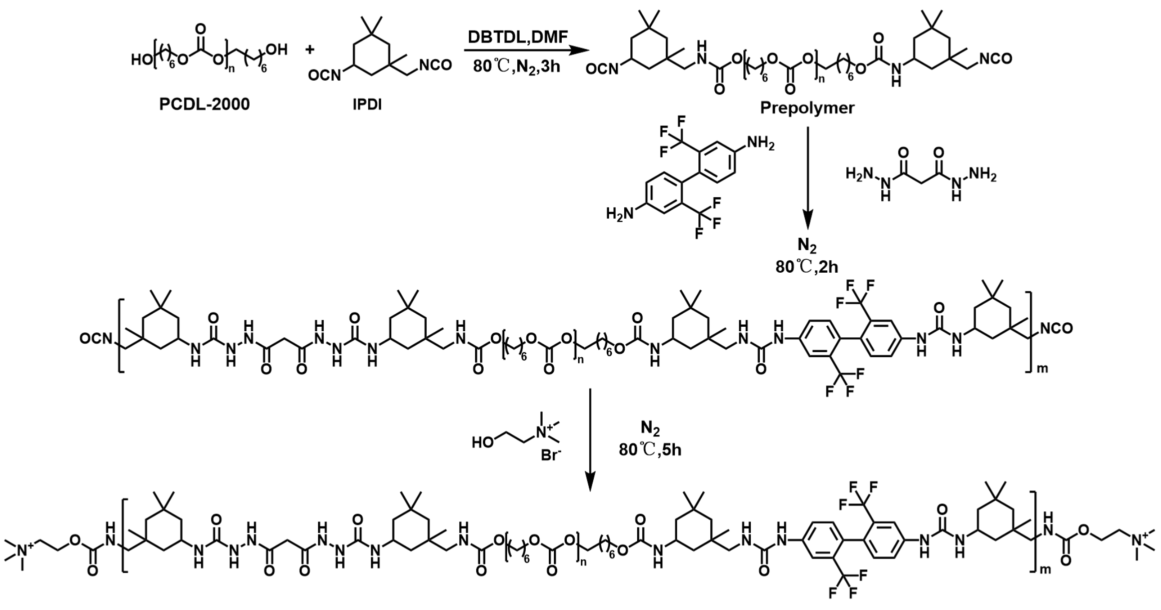

Supplementary Fig .1 Polymer design. The synthesis route of PUFT$_x$.

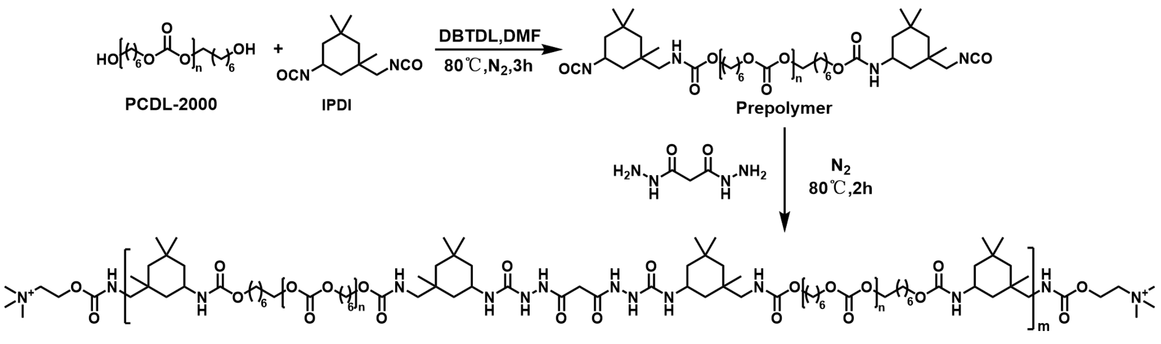

Supplementary Fig .2 Polymer design. The synthesis route of PUU.

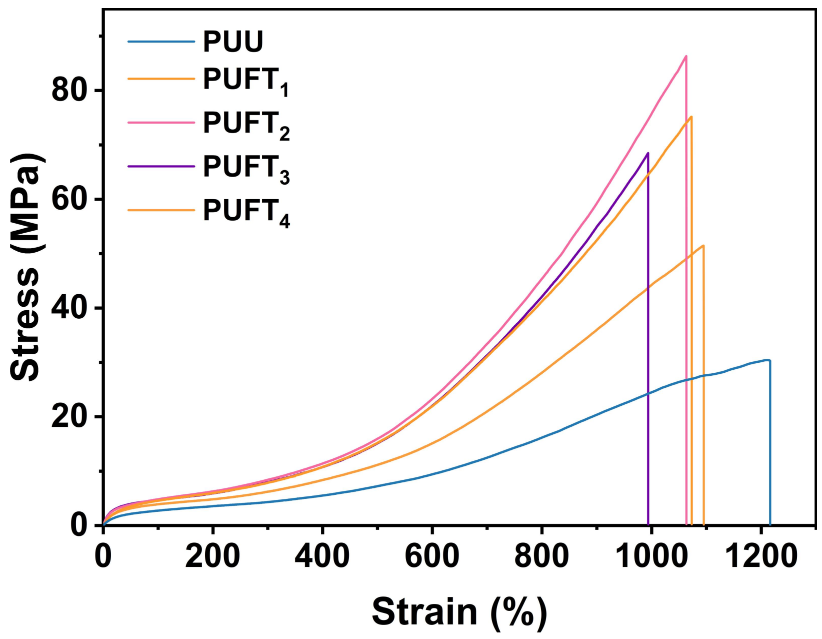

Supplementary Fig. 3 Tensile tests at room temperature. Stress–strain curves of PUU and PUFT$_x$.

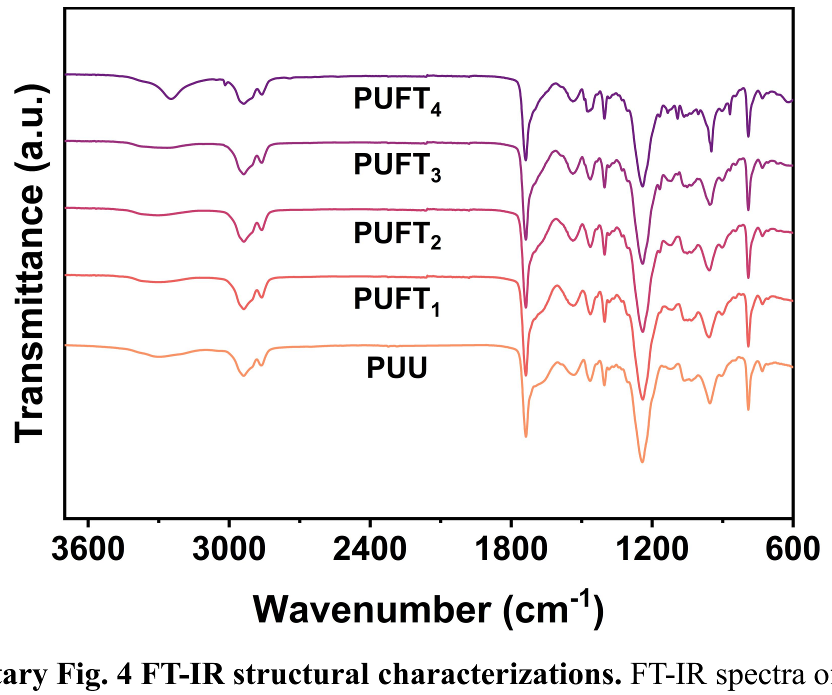

Supplementary Fig. 4 FT-IR structural characterizations. FT-IR spectra of PUFT$_x$.

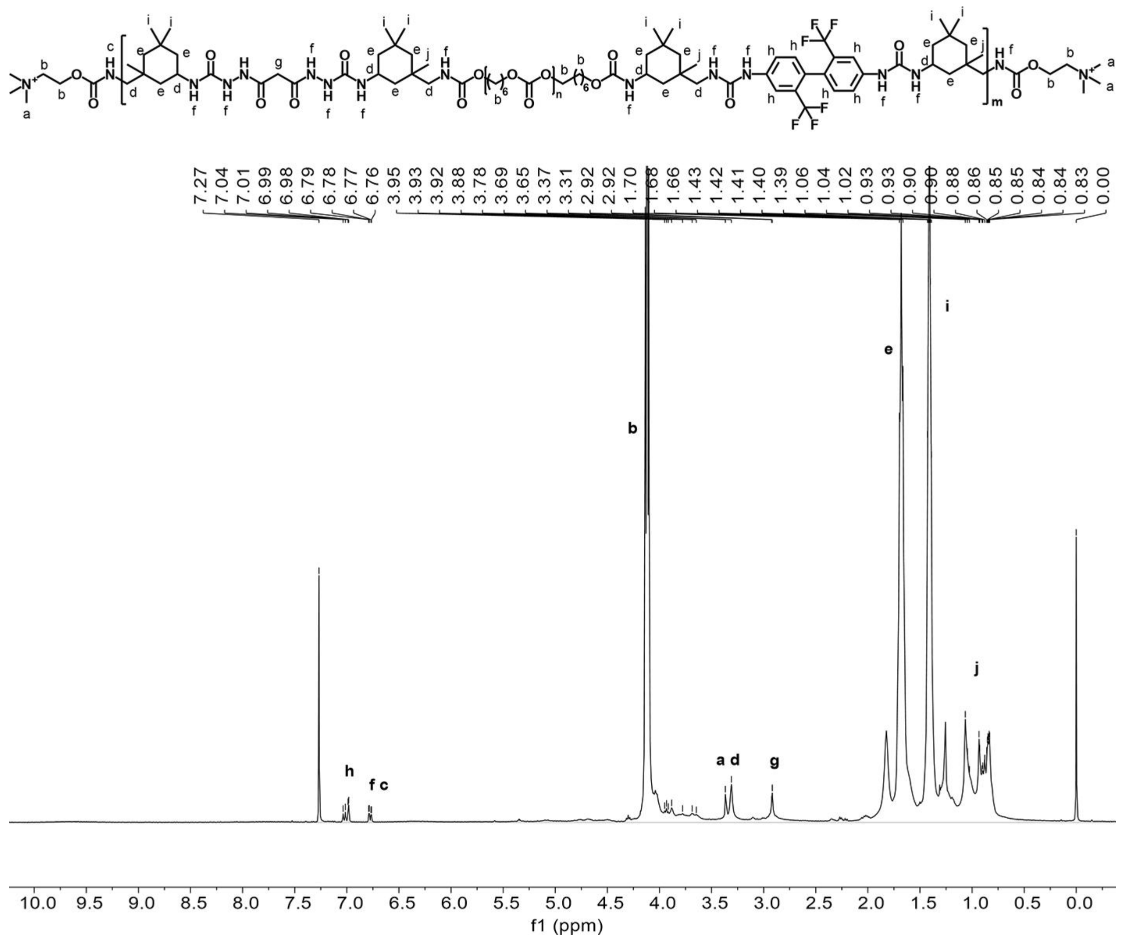

Supplementary Fig.5 NMR structural characterizations. ^{1}H NMR spectrum of $PUFT_2$ in $CDCl_3$.

^{1}H NMR (400 MHz, $CDCl_3$) δ 6.99 (m, H-h), 6.79 (d, J = 2.5 Hz, H-f), 6.77 (m, H-c), 4.12 (m, H-b), 3.37 (s, H-a), 3.31 (s, H-d), 3.03 – 2.87 (m, H-g), 1.68 (m, H-e), 1.46 – 1.22 (s, H-i), 1.16 – 0.70 (m, H-j).

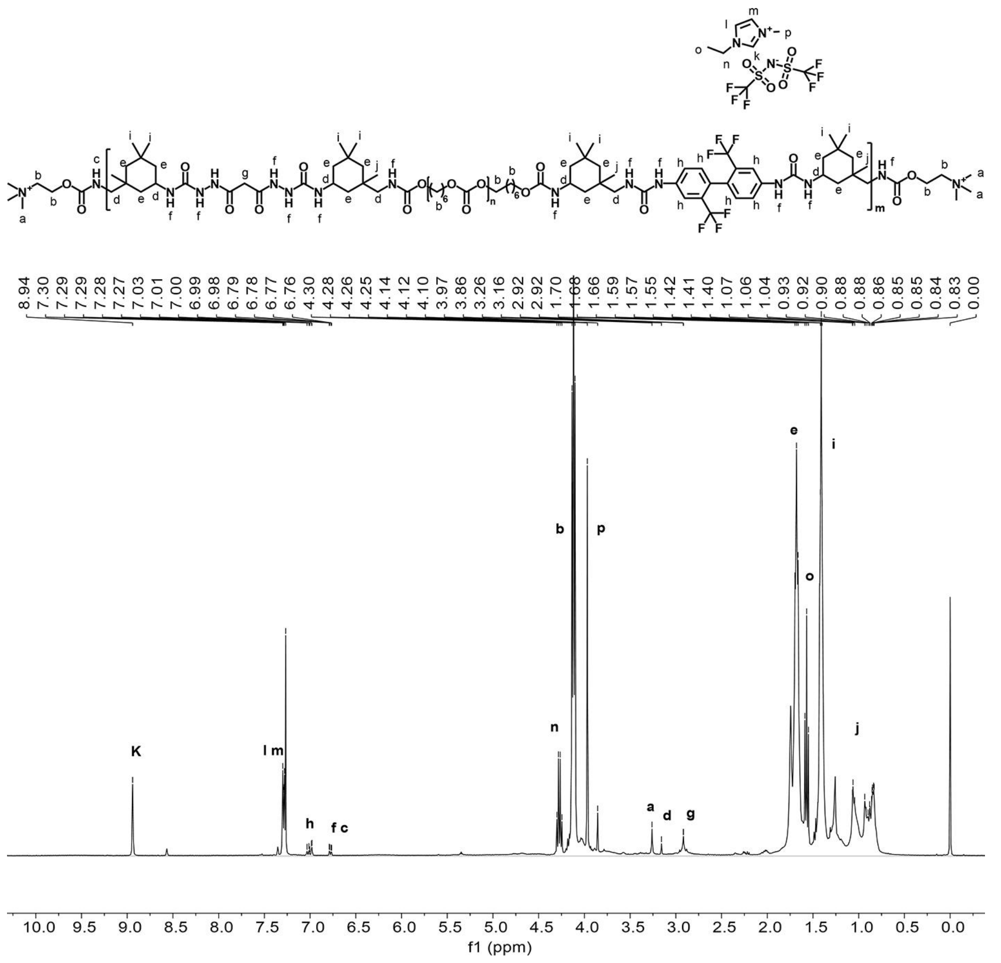

Supplementary Fig.6 NMR structural characterizations. ^{1}H NMR spectrum of PUFT$_2$-50%IL in $CDCl_3$.

^{1}H NMR (400 MHz, $CDCl_3$) δ 8.94 (s, H-k), 7.30 (d, J = 1.8 Hz, H-l), 7.28 (d, J = 1.8 Hz, H-m), 7.09 – 6.94 (m, H-h), 6.79 (d, J = 2.4 Hz, H-f), 6.77 (d, J = 2.6 Hz, H-c), 4.27 (q, J = 7.4 Hz, H-n), 4.12 (m, H-b), 3.97 (s, H-p), 3.26 (s, H-a), 3.16 (m, H-d), 2.97 – 2.86 (m, H-g), 1.67 (m, H-e), 1.57 (t, J = 7.4 Hz, H-o), 1.41 (s, H-i), 1.11 – 0.77 (m, H-j).

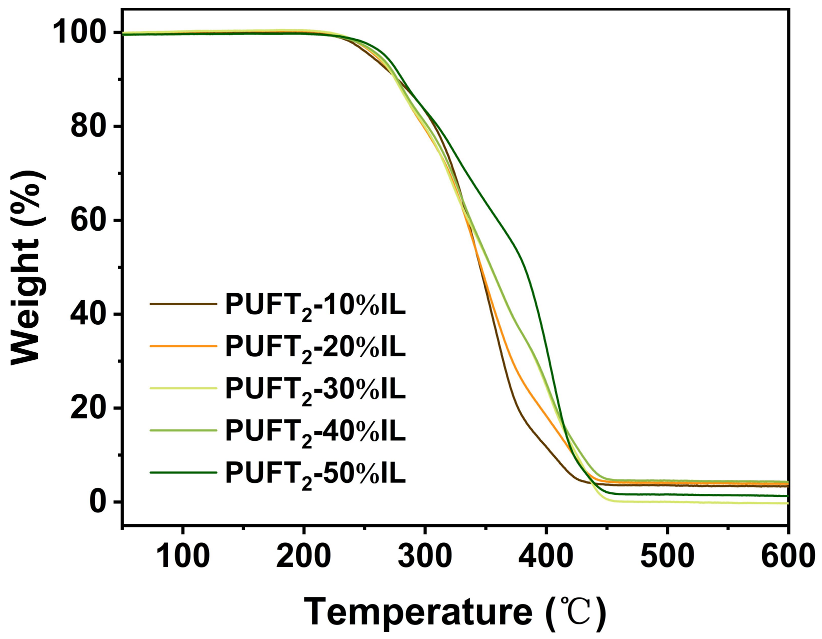

Supplementary Fig .7 Thermo-gravimetric analysis. TGA curves of $PUFT_2$-*y*IL.

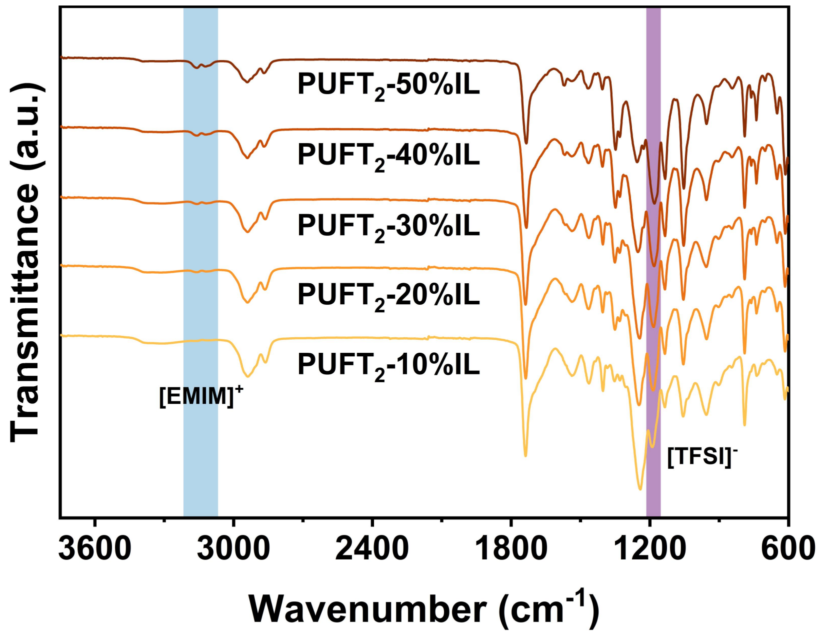

Supplementary Fig. 8 FT-IR structural characterizations. FT-IR spectra of $PUFT_2$-*y*IL.

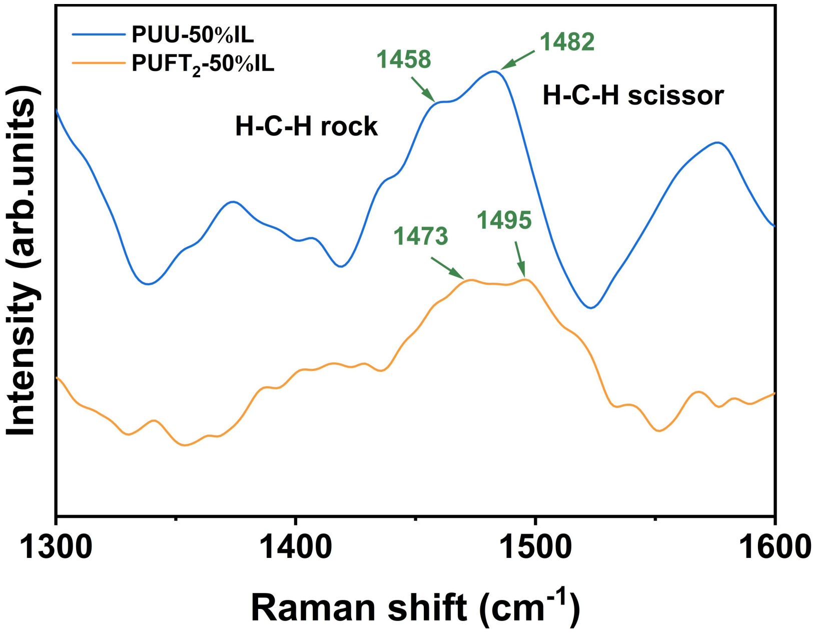

Supplementary Fig. 9 Raman shows the offset of the two characteristic peaks. Raman spectra of PUU-50%IL and $PUFT_2$-50%IL.

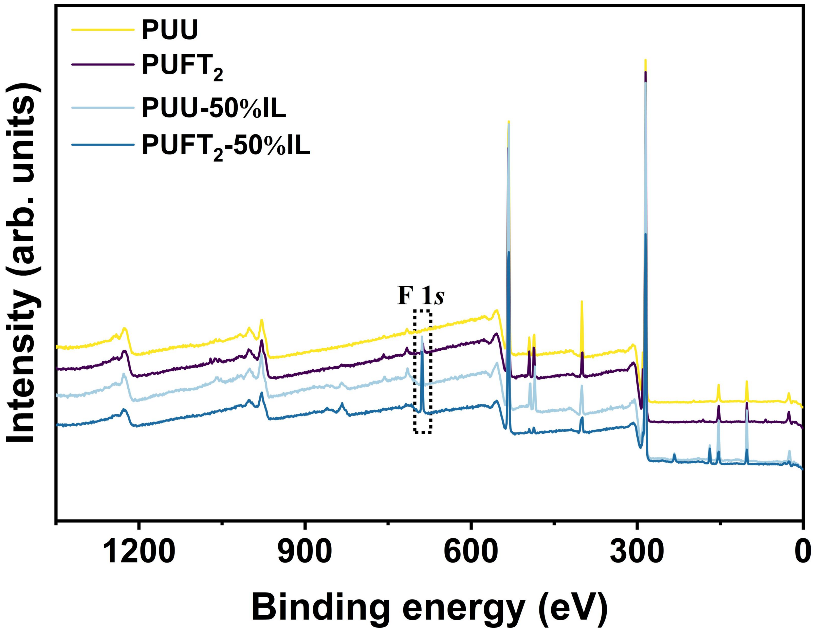

Supplementary Fig. 10 XPS shows the characteristic peak of fluorine. XPS spectra of PUU, $PUFT_2$, PUU-50%IL and $PUFT_2$-50%IL.

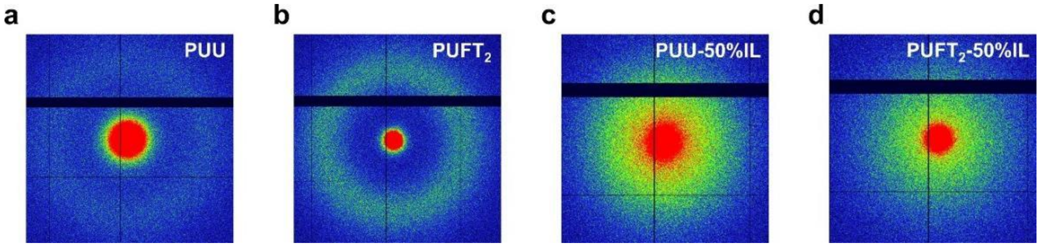

Supplementary Fig.11 2D SAXS maps. (a) PUU, (b) $PUFT_2$, (c) PUU-50%IL and (d) $PUFT_2$-50%IL.

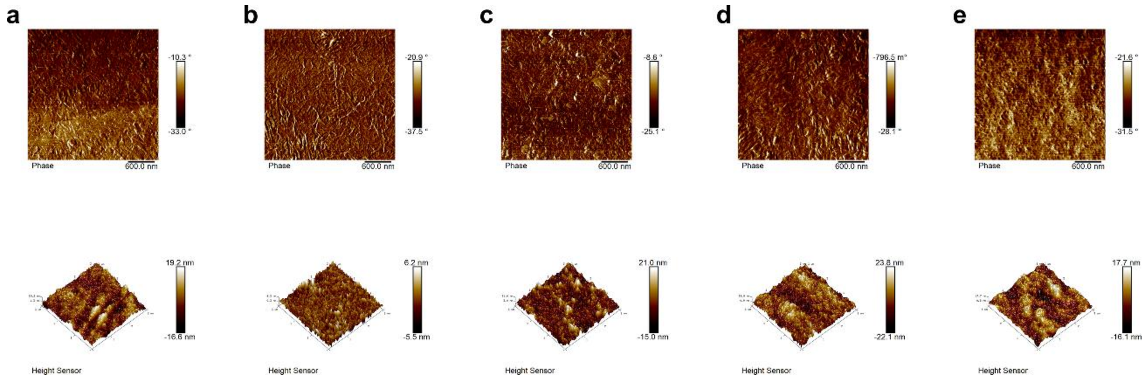

Supplementary Fig.12 AFM phase images. (a) $PUFT_2$-10%IL, (b) $PUFT_2$-20%IL, (c) $PUFT_2$-30%IL, (d) $PUFT_2$-40%IL and (e) $PUFT_2$-50%IL.

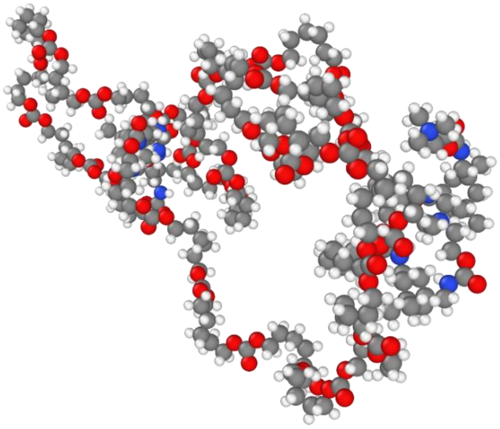

Supplementary Fig.13 Snapshots showing the MD simulations. The model of the optimized PUU polymer.

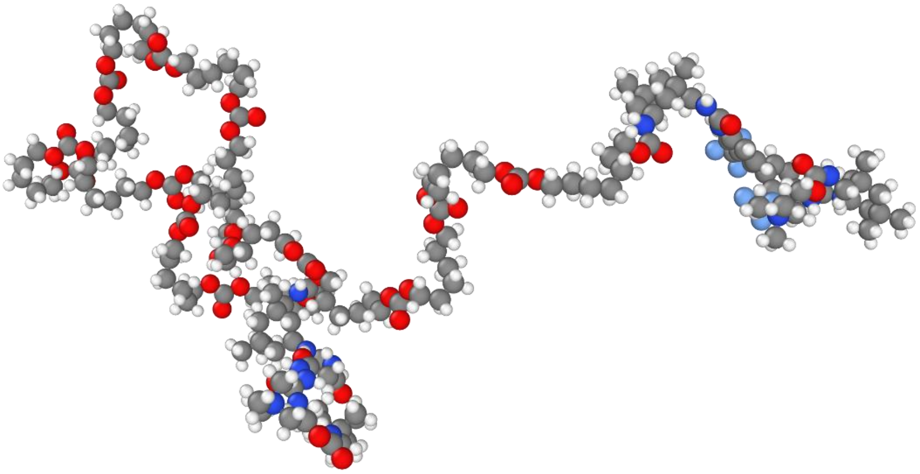

Supplementary Fig.14 Snapshots showing the MD simulations. The model of the optimized PUFT polymer.

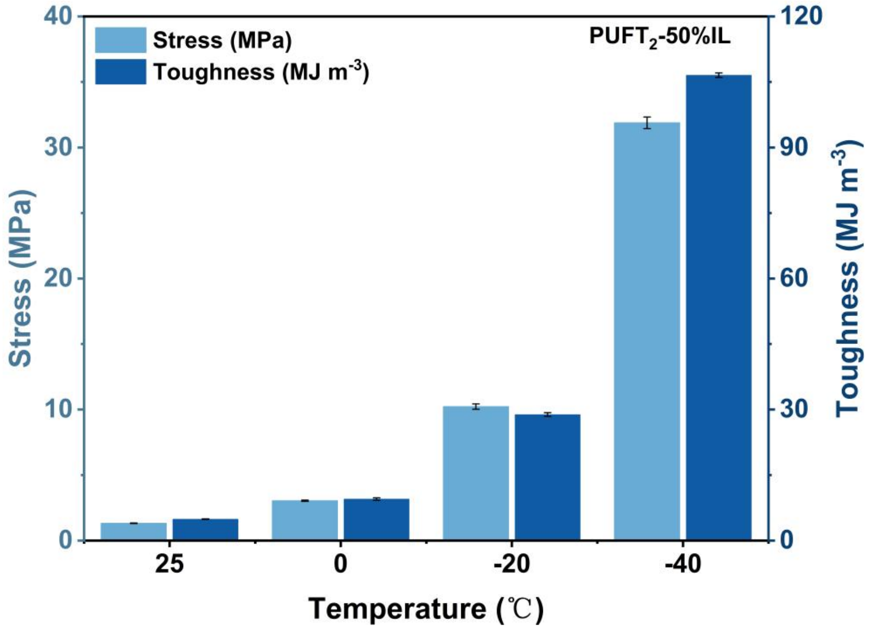

Supplementary Fig.15 The results of tensile tests at low temperatures. Tensile strengths and toughnesses of $PUFT_2$-50%IL at different temperatures. Data are presented as mean values +/- SD (n = 3).

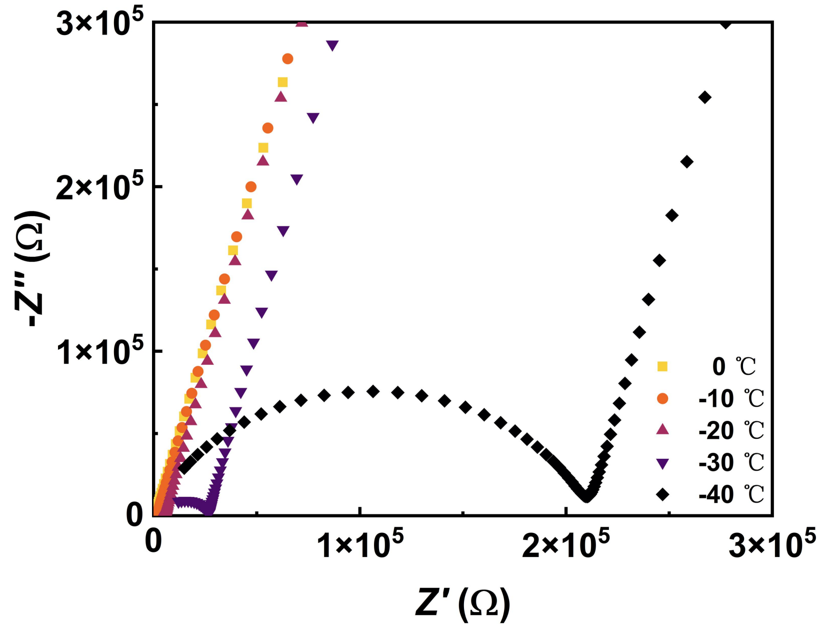

Supplementary Fig.16 Measurement of electrochemical impedance spectroscopy at low temperatures. Nyquist plot of $PUFT_2$-50%IL at different temperatures.

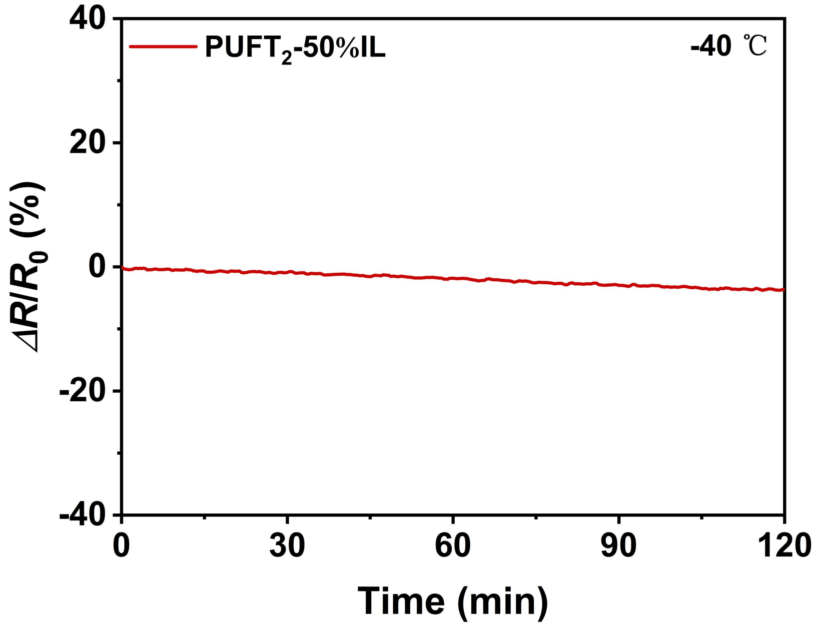

Supplementary Fig.17 Testing for resistance stability at low temperatures. $\Delta R/R_0$ of $PUFT_2$-50%IL at -40 °C for 120 minutes.

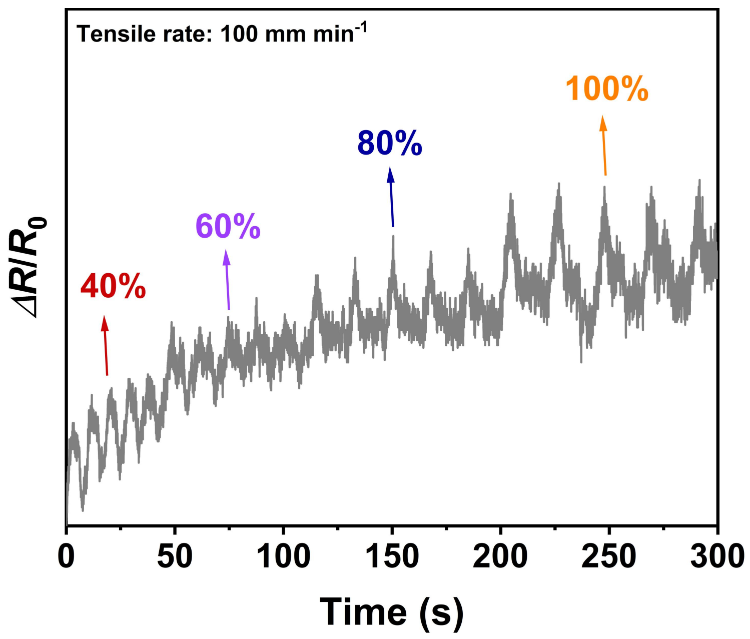

Supplementary Fig. 18 Testing the changes in resistance under continuous varying strain conditions. $\Delta R/R_0$ of $PUFT_2$-50%IL under 40–100% strain.

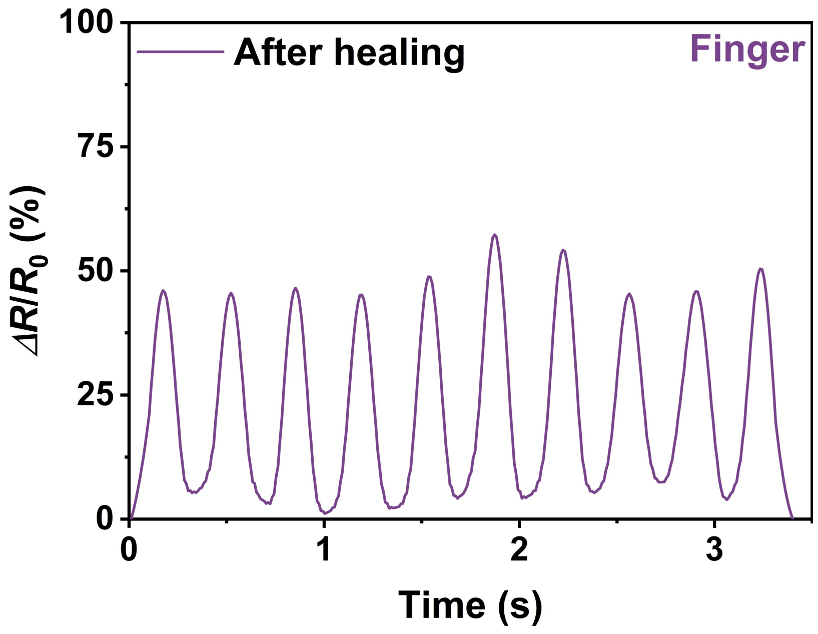

Supplementary Fig.19 Sensor performance tests for finger bending after healing. $\Delta R/R_0$ of $PUFT_2$-50%IL after healing for 1 hour during finger bending.

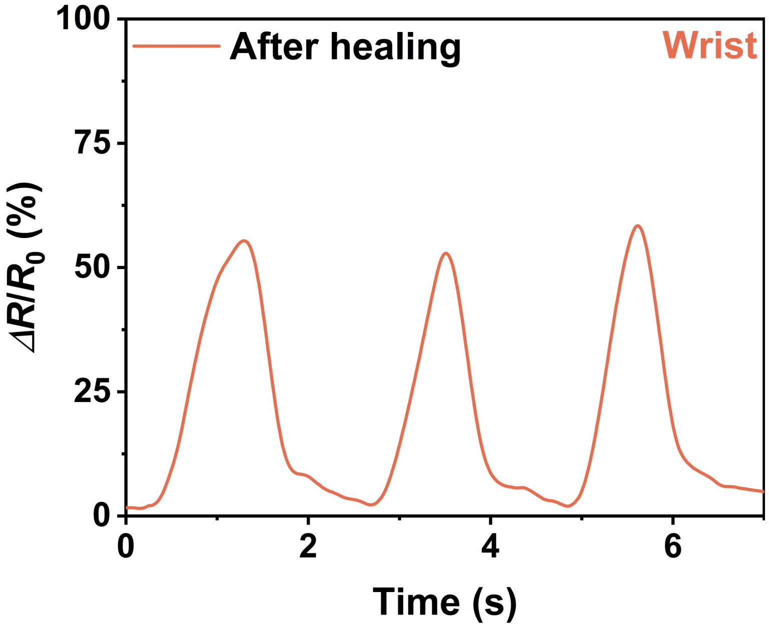

Supplementary Fig.20 Sensor performance tests for wrist bending after healing. $\Delta R/R_0$ of $PUFT_2$-50%IL after healing for 1 hour due to wrist flexion.

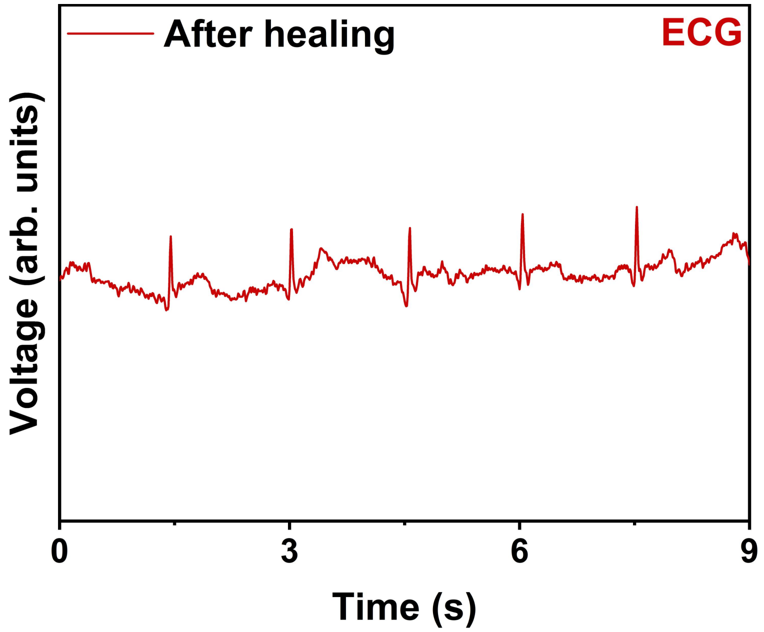

Supplementary Fig.21 ECG sensing tests after healing. ECG signals detected with the $PUFT_2$-50%IL electrode after healing for 1 hour.

Supplementary Tables

Sample	Tensile strength (MPa)	Elongation at Break (%)	Toughness (MJ m^{-3})
$PUFT_2$-10%IL	51.0 ± 0.6	1118 ± 32	166.8 ± 5.0
$PUFT_2$-20%IL	23.3 ± 0.4	1096 ± 17	82.0 ± 4.7
$PUFT_2$-30%IL	13.9 ± 0.3	1077 ± 21	49.6 ± 0.9
$PUFT_2$-40%IL	4.07 ± 0.04	1010 ± 14	20.8 ± 0.4
$PUFT_2$-50%IL	1.30 ± 0.02	936 ± 12	6.58 ± 0.05

Tensile rate:10 mm min^{-1}

Supplementary Table 1. Mechanical data of $PUFT_2$-*y*IL.

Sample	Ionic Conductivity (S cm^{-1})
$PUFT_2$-10%IL	1.04×10^{-6}
$PUFT_2$-20%IL	4.80×10^{-6}
$PUFT_2$-30%IL	1.34×10^{-5}
$PUFT_2$-40%IL	6.77×10^{-5}
$PUFT_2$-50%IL	1.34×10^{-4}

Supplementary Table 2. Ionic conductivity of $PUFT_2$-*y*IL.

**Temperatures (℃)**	**Tensile strength (MPa)**	**Elongation at Break (%)**	**Toughness (MJ m^{-3})**
0	3.04 ± 0.05	1092 ± 14	9.5 ± 0.3
-20	10.0 ± 0.2	931 ± 15	28.8 ± 0.4
-40	31.9 ± 0.4	891 ± 18	106.5 ± 0.5

Tensile rate:10 mm min^{-1}

Supplementary Table 3. Mechanical data of $PUFT_2$-50%IL at different temperatures.

**Sample**	**PCDL （mmol）**	**IPDI （mmol）**	**MDH （mmol）**	**BBD （mmol）**	**TLB （mmol）**
PUU	5	10	5	0	0
$PUFT_1$	5	10	4	0.5	1
$PUFT_2$	5	10	3	1	2
$PUFT_3$	5	10	2	1.5	3
$PUFT_4$	5	10	1	2	4

Supplementary Table 4. The molar ratio of the raw materials of PUU and $PUFT_x$.

Supplementary References

1 Frisch, M. J. *et al.* Gaussian 09 Revision A.1. Gaussian Inc. (2009).

2 Chong, D. P. *Recent Advances in Density Functional Methods*.

3 Grimme, S., Ehrlich, S. & Goerigk, L. Effect of the damping function in dispersion corrected density functional theory. *J. Comput. Chem.* **32**, 1456-1465, doi:10.1002/jcc.21759 (2011).

4 Clark, T., Chandrasekhar, J., Spitznagel, G. W. & Schleyer, P. V. R. Efficient diffuse function-augmented basis sets for anion calculations. III. The 3-21+G basis set for first-row elements, Li–F. *J. Comput. Chem.* **4**, 294-301, doi:https://doi.org/10.1002/jcc.540040303 (1983).

5 Frisch, M. J., Pople, J. A. & Binkley, J. S. Self-consistent molecular orbital methods 25. Supplementary functions for Gaussian basis sets. *The Journal of Chemical Physics* **80**, 3265-3269, doi:10.1063/1.447079 (1984).

6 Krishnan, R., Binkley, J. S., Seeger, R. & Pople, J. A. Self-consistent molecular orbital methods. XX. A basis set for correlated wave functions. *The Journal of Chemical Physics* **72**, 650-654, doi:10.1063/1.438955 (1980).

7 Thompson, A. P. *et al.* LAMMPS - a flexible simulation tool for particle-based materials modeling at the atomic, meso, and continuum scales. *Comput. Phys. Commun.* **271**, 108171, doi:https://doi.org/10.1016/j.cpc.2021.108171 (2022).

8 Wang, J., Wolf, R. M., Caldwell, J. W., Kollman, P. A. & Case, D. A. Development and testing of a general amber force field. *J. Comput. Chem.* **25**, 1157-1174, doi:https://doi.org/10.1002/jcc.20035 (2004).
