# Supplementary material for: Shape-memory polyurethanes for polar wearables with ultrasensitive multi-monitoring
Source: Nat Commun. 2025 Dec 11;16:11329. doi: 10.1038/s41467-025-66422-3 (PMC12722725; doi:10.1038/s41467-025-66422-3)
Supplement: Supplementary file 2 — Description of Additional Supplementary Files [file 41467_2025_66422_MOESM2_ESM.doc]

**Description of Additional Supplementary Files**

Supplementary Movie 1

Description：Demonstration of the damping performance, capable of protecting glass from impact by a 100g steel ball falling from 50 cm height.

Supplementary Movie 2

Description：Demonstration of the shape memory effect.

Supplementary Movie 3

Description：Demonstration of the smart wearable based on shape memory effect.
